# Acquired immunodeficiency associated with thymoma: a case report

**DOI:** 10.1186/s12885-019-5980-y

**Published:** 2019-08-02

**Authors:** Takahisa Kawamura, Tateaki Naito, Haruki Kobayashi, Kazuhisa Nakashima, Shota Omori, Kazushige Wakuda, Akira Ono, Hirotsugu Kenmotsu, Haruyasu Murakami, Masahiro Endo, Toshiaki Takahashi

**Affiliations:** 1 Division of Thoracic Oncology, Shizuoka Cancer Center Hospital, 1007 Shimonagakubo, Nagaizumi-cho, Sunto-gun, Shizuoka, 411-8777 Japan; 2 Division of Diagnostic Radiology, Shizuoka Cancer Center Hospital, Shizuoka, Japan

**Keywords:** Thymoma, Radiotherapy, Immunodeficiency, Meningitis, Good syndrome

## Abstract

**Background:**

Acquired immunodeficiency associated with thymoma is a rare disorder. Here we reported a case of acquired immunodeficiency with thymoma, with an unusual pattern of low CD4^+^ count with normal gammaglobulin levels.

**Case presentation:**

A 70-year-old man presented to the emergency room of our hospital with a high-grade fever, headache, and nausea. He had a five-year history of unresectable thymoma treatment, including several cytotoxic regimens. He had received thoracic palliative radiotherapy 2 months prior to the emergent visit. During the previous month, he had experienced multiple febrile episodes, dry cough, fatigue, weight loss, and watery diarrhea. Upon admission, he had a high-grade fever, nausea, and immobility. Physical examination revealed indistinct consciousness, neck stiffness, and oropharyngeal candidiasis. Both cerebrospinal fluid and blood cultures yielded multiple short chains of Gram-positive rods later identified as *Listeria monocytogenes*, so he was diagnosed with *Listeria* meningitis. Intravenous administration of antibiotics was initiated, and the patient fully recovered and was discharged. Additional examination found normal immunoglobulin levels. Peripheral-blood cell counts revealed low CD4^+^ cell count (108 CD4^+^ cells/μl). His CD4^+^ cell count remained low after discharge.

**Conclusions:**

Our findings suggest that physicians need to be aware of severe infections due to immunodeficiency with thymoma.

## Background

Acquired immunodeficiency associated with thymoma, represented by Good syndrome, is a rare disorder. Good syndrome was first described more than 60 years ago, characterized by humoral immunodeficiency of hypogammaglobulinemia often with the onset after thymectomy [1]. Here we report a case of acquired cellular immunodeficiency that was induced immediately after radiotherapy for thymoma, with an atypical pattern of low CD4^+^ count with normal gammaglobulin levels.

## Case presentation

**Fig. 1:**
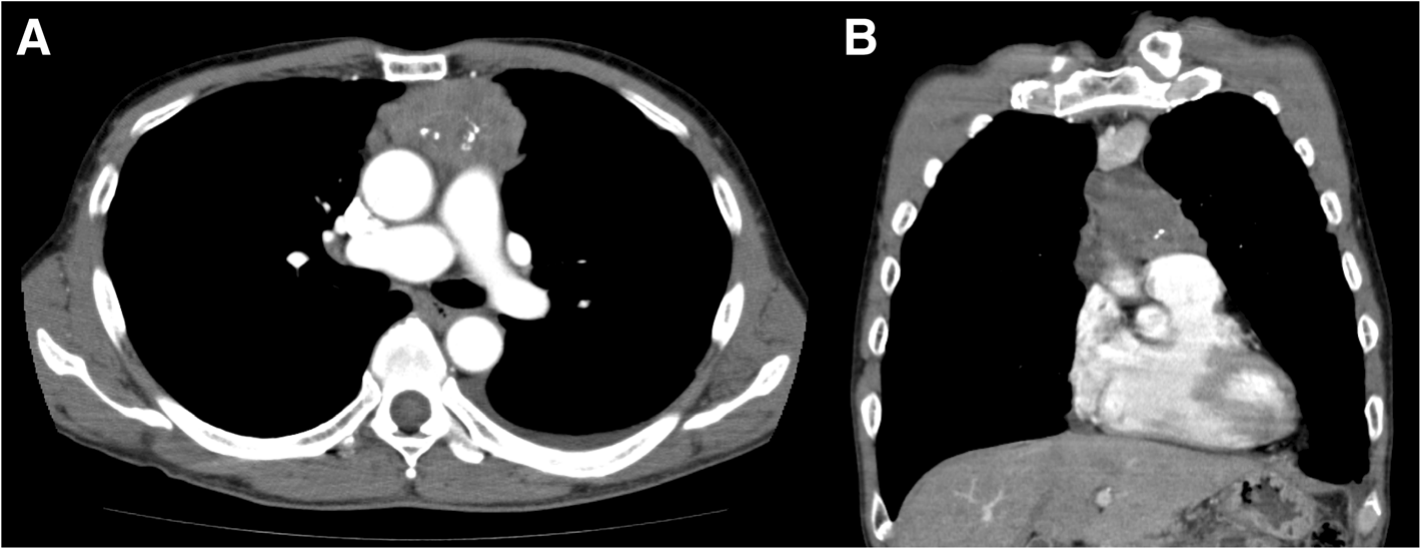
Computed tomography imaging of the chest at diagnosis. Transverse (**a**) or coronal (**b**) planes in a mediastinal window

**Fig. 2:**
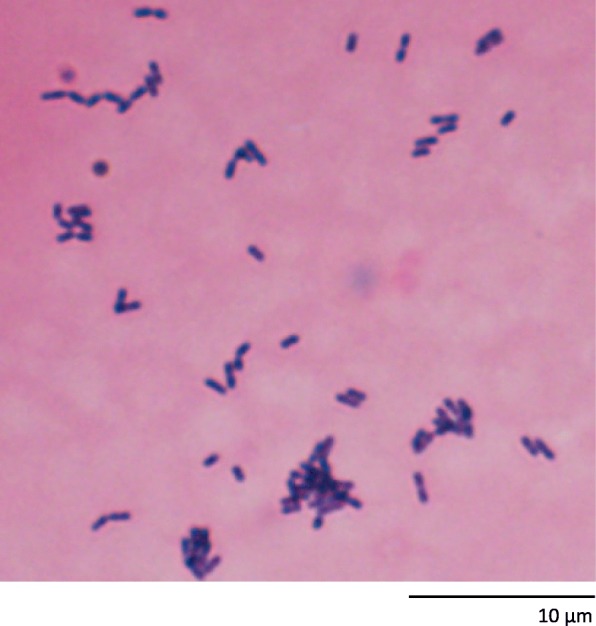
Blood culture findings upon emergent admission

A 70-year-old man presented to the emergency room of Shizuoka Cancer Center with a high-grade fever, headache, and nausea. He had a five-year history of unresectable thymoma (Masaoka stage IVa; Fig. 1) treatment, including cisplatin and amrubicin, amrubicin monotherapy, and a phosphoinositide 3-kinase/mammalian target of rapamycin inhibitor which was discontinued due to tumor progression 12 months prior. Two months prior, he had received thoracic radiotherapy (40 Gy with 20 fractions) for palliation of chest pains due to multiple pleural disseminated lesions. During the previous month, he had experienced multiple febrile episodes, dry cough, fatigue, weight loss, and watery diarrhea. Upon admission, he had a fever (≥40 °C), nausea, and immobility. Physical examination revealed indistinct consciousness, neck stiffness, and oropharyngeal candidiasis. The patient’s white blood cell count was 15,380 cells/μl with 91% granulocytes, and biochemical examination revealed elevated AST/ALT levels (81 and 153 IU/L, respectively). Both cerebrospinal fluid and blood cultures yielded multiple short chains of Gram-positive rods later identified as *Listeria monocytogenes*, so he was diagnosed with *Listeria* meningitis (Fig. 2). Intravenous administration of vancomycin (1 g/12 h) and ceftriaxone (2 g/12 h) was initiated, followed by ampicillin (2 g/4 h) and gentamicin (80 mg/8 h) for three weeks. The patient fully recovered and was discharged.

Additional examination found normal levels of total IgG (1148 mg/dL), IgM (57 mg/dL), and IgA (156 mg/dL). Flowcytometric analysis of peripheral-blood cells disclosed 528 lymphocytes/μl (normal range, 1000–3100 lymphocytes/μl), 108 CD4^+^ cells/μl (normal range, 320–1900 cells/μl), 129 CD8^+^ cells/μl with a CD4^+^/CD8^+^ ratio of 0.84 (normal range, 0.40–2.30), and 2% B cells (11 cells/μl). Tests for antibodies to human immunodeficiency virus type 1 (HIV) and human T-lymphotropic virus type 1 were negative. The patient was diagnosed with cellular immunodeficiency due to thymoma. His CD4^+^ cell count has remained < 150 cells/μl during 15-month follow up after discharge (after 3 months, 79 cells/μl; 6 months, 107 cells/μl; 9 months, 146 cells/μl; 12 months, 132 cells/μl).

## Discussion and conclusions

To our knowledge, few reports have discussed immunodeficiency identified after radiotherapy for thymoma. Patients with immunodeficiency often exhibit respiratory tract infections and chronic diarrhea [2–4]. Thus, the patient’s frequent diarrhea might have been an early manifestation. Furthermore, he had oral candidiasis and *Listeria* meningitis, which may reflect a dysfunction in T-cell mediated immunity [5].

As originally described, Good Syndrome is hypogammaglobinemia in patients with underlying thymoma. However, our peripheral blood examination revealed a rarely reported pattern of low CD4^+^ count with normal gammaglobulin levels, more typical of HIV infection [6].

The proportion of peripheral CD45RA^+^ T cells reportedly decreases after thymoma resection [7]. Additionally, adults have deficiencies in thymus-dependent CD4^+^ T lymphocyte regeneration; therefore, rapid T-cell regeneration requires residual thymic function in patients receiving high-dose chemotherapy [8]. One possible explanation is the large volume reduction caused by radiotherapy also induces similar systemic immune response, leading to acquired immunodeficiency [9]. However, a direct relationship between radiotherapy and immunodeficiency was not proven because the CD4+ cell count before radiotherapy was not measured in this case. A previous report suggested that cellular immunodeficiency associated with thymoma was not uncommon, but is frequently overlooked [10]. In this case, it was possible that the patient already had undiagnosed cellular immunodeficiency before radiotherapy.

In thymoma cases, CD4 levels may have to be measured frequently over time, irrelevant of whether the baseline immunoglobulin levels are within the range or not. Correct diagnosis would provide treatment of the paraneoplastic Good syndrome to avoid complications induced by therapies such as radiotherapy, chemotherapy, and surgery.

In conclusion, we reported a case of acquired cellular immunodeficiency in a patient with thymoma. Our findings suggest that physicians need to be aware of severe infections due to immunodeficiency in patients with thymoma.

## Data Availability

All data in this report is confidential patient information which is kept as part of the medical record at the site of care.

## Abbreviations

Gy: Gray
HIV: Human immunodeficiency virus
IU: International unit
